# Single- versus double-bundle patellar graft insertion for isolated MPFL reconstruction in patients with patellofemoral instability: a systematic review of the literature

**DOI:** 10.1007/s00402-020-03376-9

**Published:** 2020-02-21

**Authors:** Filippo Migliorini, Andromahi Trivellas, Giorgia Colarossi, Jörg Eschweiler, Markus Tingart, Björn Rath

**Affiliations:** 1 Department of Orthopaedics, RWTH Aachen University Clinic, Pauwelsstraße 30, 52074 Aachen, Germany; 2 Department of Orthopaedics, David Greffen School of Medicine at UCLA, Los Angeles, CA USA; 3 Department of Cardiosurgery, RWTH Aachen University Clinic, Aachen, Germany

**Keywords:** Patellofemoral instability, Dislocation, MPFL, Reconstruction, Graft, Single bundle, Double bundle

## Abstract

**Introduction:**

The MPFL reconstruction is performed either via a single-bundle (SB) or double-bundle (DB) procedure. The purpose of this study is to perform a systematic review comparing SB versus DB graft for recurrent patellofemoral instability, to summarize current evidence, and to clarify the role of both techniques. We focused on clinical scores, physical examination, complications, revision surgeries, and failures.

**Material and methods:**

In May of 2019 the main online databases were accessed. All the clinical studies treating isolated MPFL reconstruction for patellofemoral instability through a single and/or double-bundle graft were enrolled in the present systematic review. Only articles reporting primary isolated MPFL reconstruction, reporting a minimum of 12-months follow-up were considered for inclusion.

**Results:**

The scores of interest were in favour of the DB cohort: Kujala (+ 3.2, *P* = 0.03), Lysholm (+ 5.1, *P* = 0.001), Tegner (+ 0.3, *P* = 0.2), IKDC (+ 5.4, *P* = 0.01), VAS (+ 0.8, *P* = 0.3), ROM (+ 9.96, *P* = 0.04). In the DB graft, a reduction of overall complications (OR 0.59; *P* = 0.1), further surgeries (OR 0.64; *P* = 0.12) and re-dislocations (OR 0.61; *P* = 0.16) was observed. The SB group reported a reduction in the post-operative apprehension test (OR 2.42; *P* = 0.24).

**Conclusion:**

Current study support the use of double-bundle tendon graft for isolated MPFL reconstruction in selected patients with recurrent patellofemoral instability.

## Introduction

Patellofemoral instability is a multifactorial disorder that affects young active patients [1]. Regardless of the cause of dislocation, up to 94% of knees reported damage to the medio patellofemoral ligament (MPFL) [2]. The MPFL is the most important restraint to lateral displacement of the patella during the first 30° of knee flexion [3]. In up to 44% of patients, conservative treatment resulted in recurrent patellar dislocation [4]. Surgical reconstruction of the MPFL reported excellent outcomes in terms of patient satisfaction, quality of life, and knee scores, in addition to a very low rate of re-dislocations and complications [5]. It has been supposed that isolated MPFL reconstruction may even achieve satisfactory results in patients with mild to moderate patho-anatomical risk factors thus avoiding more invasive procedures [6]. During MPFL reconstruction, for a correct femoral ligament insertion, the radiographic method described by Schöttle et al. [7]. For the patellar ligament insertion, both medial retinaculum and native MPFL are dissected from the patella, leaving the capsule layer intact. Patellar insertion can be performed either via a single (SB) or a double-bundle (DB) procedure. Several techniques are described for MPFL reconstruction either via single and double bundles. The DB was developed to simulate anatomical stress distribution [8] and was designed to reduce the rate of failures and complications [9, 10]. However, results are controversial, and debates are still ongoing [11–14].

Hence, the purpose of this study is to perform a systematic review comparing SB versus DB grafts for recurrent patellofemoral instability, to summarize the current evidence, and clarify the role of both the techniques. We focused on the clinical scores, physical examination, complications, revision surgeries and failures.

## Material and methods

### Search strategy

This systematic review was conducted according to the Preferred reporting items for systematic reviews and meta-analyses (PRISMA) [15]. A preliminary protocol was drafted to outline the search parameters:

- P (population): recurrent patellofemoral instability;
- I (intervention): isolated MPFL reconstruction;
- C (comparison): double versus single bundle tendon graft;
- O (outcome): clinical scores and examination, complications, surgical revision and failure.

### Literature search

The search was conducted by two independent authors (FM, JE) in May 2019. The following databases were accessed: Pubmed, Scopus, Embase, and Google scholar. The following keywords were used in combination: *patellofemoral*,* recurrent*,* patellar*,* instability*,* dislocation*,* syndrome*,* luxation*,* MPFL*,* reconstruction*,* isolated*,* tear*,* rupture*,* graft*,* single*,* double*,* bundle*,* semitendinosus*,* gracilis*,* hamstring*,* synthetic*,* failure*,* apprehension test*,* Kujala*,* Lysholm.* If the title and subsequent abstract matched the topic, the full-text of the article was accessed. The bibliographies of the included articles were also screened to find potentially missed articles. Disagreements between the authors were debated and mutually resolved.

### Eligibility criteria

All articles treating MPFL reconstruction for recurrent patellofemoral instability through a single and/ or double-bundle graft were enrolled in the present systematic review. According to the authors language capabilities, only articles in Italian, German, English, French, Spanish were included. According to the Oxford Centre of Evidenced-Based Medicine [16], articles with the level of evidence I to IV were considered for inclusion. Only articles reporting primary MPFL reconstruction were included in the present study. Only articles reporting isolated MPFL reconstruction were considered. Comments, techniques, editorials, letters, protocols, expert opinion, and guidelines were excluded. Biomechanical, animal, and cadaveric studies were also excluded. Only articles reporting a minimum of 12-months follow-up were considered. Studies treating patellofemoral instability after total knee arthroplasty were also rejected. Only articles that reported quantitative data concerning the endpoints of interest were included. Disagreements between the authors were debated and mutually resolved.

### Outcomes of interest

Two independent authors (FM, JE) recorded the following data: study generalities (author, year, type of study), patient baselines (mean age, duration of the follow-up, time injury to surgery), type of instability (recurrent and/ or acute), presence of risk factors (trochlear dysplasia, patella alta, elevated TT-TG), and surgical graft fixation. Data concerning the following parameters were also recorded: Kujala Anterior Knee Pain Scale [17], Lysholm Knee Scoring Scale [18], Tegner Activity Scale [19], International Knee Documentation Committee (IKCD) [20], visual analogic scale (VAS) and knee range of motion (ROM). In addition, clinical examination, complications, further re-operations, and failures were recorded.

### Methodological quality assessment

For the methodological quality assessment, the PEDro score was performed. This score was evaluated by two independent authors (FM, JE). This score analyses the included articles under several points of view: statement of the eligibility criteria, allocation, randomization and blinding methods, duration of follow-up, intention to treat, point estimates, and variability. The final value ranks from 0 (poor quality) to 10 (excellent quality). Value > 6 points are considered acceptable.

### Statistical analysis

The statistical analyses were performed through the IBM SPSS Statistic software. Continuous data were analysed via arithmetic mean, standard deviation and range of interval. Binary data were analysed through the odd ratio (OR) effect measure. The confidence interval was set at 95% in all the comparisons. The unpaired t-test was performed in all the comparisons. A *P* value < 0.05 was considered statistically significant.

## Results

### Search result

**Fig. 1:**
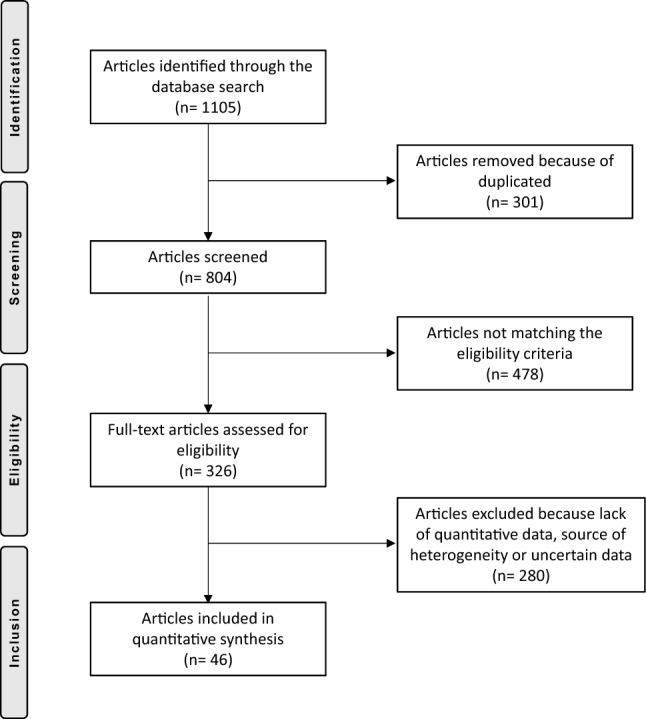
PRISMA flow-chart of the literature search

From the initial search, we obtained a total of 1105 articles. From this pool, 301 were rejected due to duplication. Another 478 articles were excluded due to discrepancies in meeting eligibility criteria. A further 275 articles were rejected due to the lack of quantitative data concerning the outcomes of interest. This left 51 articles for inclusion. Of them, another 6 articles were excluded due to uncertain data. Ultimately, a total of 46 studies were included, 29 performing double-bundle MPFL reconstruction, 16 using the single-bundle technique, 1 comparing both the techniques. We enrolled four randomized clinical trials (RCT), 19 prospective cohort studies (PCS), 19 retrospective cohort studies (RCS), four case series (CS). The type of the included studies according to the grafts are shown in Table 1. The flow-chart of the literature search is shown in Fig. 1.

**Table 1:** Generalities, demographic data and related PEDro scores of the included studies concerning the DB graft

Author, year	Type of study	PEDro score	Knees (*n*)	Mean age	Time injury to surgery	Mean follow-up (months)
Astur et al. 2015 [11]	RCT	8	28	28.32		60.00
Bitar et al. 2012 [21]	PCS	7	56	23.00		19.30
Christiansen et al. 2008 [22]	PCS	6	32	22.00	84.00	22.00
Csintalan et al. 2014 [23]	CS	5	56	24.30	86.40	51.00
Deie et al. 2011 [24]	RCS	5	31	22.20		39.00
Feller et al. 2014 [25]	RCS	5	26	24.40	88.80	42.00
Fink et al. 2014 [26]	PCS	7	17	21.50		12.00
Goncaives et al. 2011 [27]	PCS	6	22	28.60	141.00	26.20
Hinterwimmer et al. 2013 [28]	RCS	6	19	23.00		16.00
Kang et al. 2013 [29]	RCT	8	82	28.75		24.00
Kita et al. 2015 [30]	PCS	7	44	25.40	156.00	39.00
Krishna Kumar et al. 2014 [31]	PCS	7	30	18.00	33.53	25.00
Kumahashi et al. 2012 [32]	PCS	6	5	13.60	19.00	27.80
Kumahashi et al. 2016 [33]	PCS	7	17	22.00	61.00	45.00
Li et al. 2014 [34]	PCS	7	65	29.40		78.50
Lind et al. 2016 [35]	PCS	8	24	12.50		39.00
Lind et al. 2016 [35]	PCS	8	179	23.00		41.00
Lin et al. 2015 [36]	RCS	5	18	N/R		35.00
Lippacher et al. 2014 [37]	RCS	7	68	18.30		24.70
Ma et al. 2013 [38]	RCT	8	32	28.40	26.00	40.00
Matsushita et al. 2014 [39]	RCS	6	21	22.10		44.00
Matsushita et al. 2014 [39]	RCS	6	18	23.50		38.00
Niu et al. 2017 [40]	PCS	7	30	25.00		55.10
Panni et al. 2011 [41]	CS	5	48	25.00	12.00	33.00
Ronga et al. 2009 [42]	PCS	5	37	28.00		37.00
Sadigursky et al. 2016 [43]	PCS	7	31	29.38		12.00
Smith et al. 2014 [44]	RCS	6	21	23.00	95.00	12.00
Suganuma et al. 2016 [45]	RCS	6	18	20.70		51.60
Suganuma et al. 2016 [45]	RCS	6	28	20.30		48.00
Thaunat et al. 2007 [46]	RCS	5	23	22.00		28.00
Toritsuka et al. 2011 [47]	CS	6	20	23.80		30.00
Wang et al. 2013 [46]	RCS	7	44	26.00		48.00
Wang et al. 2016 [48]	RCS	6	26	26.30		38.20
Wantabe et al. 2008 [49]	RCS	7	29	19.00	43.20	51.60
Zhang et al. 2019 [50]	PCS	7	60	21.00	12.00	96.00

*RCT* randomized clinical trial, *PCS* prospective cohort study, *RCS* retrospective cohort study, *CS* case series

### Methodological quality assessment

The PEDro score evidenced some weakness of this systematic review. Only 9% of the articles provided randomization. None of the included articles provided blinding methods. However, the overall quality of the included papers, the follow-up duration, and the number of included patients were acceptable. In total, the PEDro score resulted in 6.34 ± 1.1 points, attesting to the good methodological quality assessment of this systematic review. The results of the PEDro score assigned for each study are shown in Tables 1 and 2.

**Table 2:** Generalities, demographic data and related PEDro scores of the included studies concerning the SB graft

Author, year	Type of study	PEDro score	Knees (*n*)	Mean age	Time surgery to injury	Mean follow-up (months)
Ahmad et al. 2009 [51]	CS	5	20	23.00		31.00
Amin et al. 2015 [52]	RCS	6	8	22.00	9.00	24.00
Astur et al. 2015 [11]	RCT	8	30	31.06		60.00
Bitar et al. 2011 [53]	RCT	8	21	NR		24.00
Calapodopulos et al. 2016 [54]	PCS	5	22	23.10		30.00
Ellera Gomes et al. 1992 [55]	RCS	5	30	28.00	90.00	39.00
Ellera Gomes et al. 2004 [56]	PCS	6	16	26.70	1.00	60.00
Gomes et al. 2008 [57]	PCS	7	12	19.30		53.00
Goyal et al. 2013 [58]	RCS	5	32	25.00		38.00
Han et al. 2011 [59]	RCS	6	59	24.30	45.60	68.40
Hiemstra et al. 2017 [60]	RCS	5	155	25.40		24.40
Howells et al. 2012 [61]	PCS	7	155	26.00		16.00
Howells et al. 2012 [61]	PCS	7	55	26.00		16.00
Nomura et al. 2000 [62]	PCS	7	27	21.00	55.20	70.80
Nomura et al. 2006 [63]	RCS	6	12	24.80	122.40	51.00
Nomura et al. 2007 [64]	RCS	5	24	22.50		142.80
Pinheiro et al. 2018 [65]	RCS	7	16	27.10		31.20
Pinheiro et al. 2018 [65]	RCS	7	21	26.40		34.80
Raghuveer et al. 2012 [66]	PCS	7	15	29.20	141.60	42.00
Sillanpaa et al. 2008 [67]	RCS	6	18	20.20		121.20
Slenker et al. 2013 [68]	RCS	6	35	20.60	57.60	21.00
Steiner et al. 2006 [69]	CS	6	34	27.00		66.50
Vavalle et al. 2015 [70]	RCS	5	16	22.00		38.00
Wang et al. 2013 [12]	RCS	7	26	23.00		48.00
Wang et al. 2010 [71]	RCS	7	28	29.00	30.00	42.00

*RCT* randomized clinical trial, *PCS* prospective cohort study, *RCS* retrospective cohort study, *CS* case series

### Patient demographic

Data from 2204 patients were obtained. The patients at the time of surgery had a mean age of 23.66 ± 3.7 years. The mean duration of the follow-up was 45.02 ± 22.1 months. 24% of the studies harvested the gracilis tendon for the reconstruction, 60% the semitendinosus. Other grafts were the adductor magnus, quadriceps, patellar, hamstring, tibialis anterior, and five synthetic tendons. In the DB group, a total of 1305 patients were enrolled, with a mean age of 23.01 ± 4.0 years. The time between the first dislocation and surgery in this group was 65.99 ± 47.5 months (Table 1). In the SB group, a total of 899 patients were enrolled, with a mean age of 24.53 ± 3.3 years. The time between the first dislocation and surgery in this group was 61.38 ± 48.3 months (Table 2). No significant discrepancies between the two groups concerning age and time injury to surgery were detected (*P* = 0.07 and *P* = 0.4, respectively).

### Outcomes of interest

The DB group reported a mean Kujala score of 89.84% (range 71.0–97.7, SD 5.9), mean Lysholm score of 91.79% (range 87.9–96.4, SD 2.7), mean Tegner score of 5.36 (range 4.0–7.8, SD 1.1), mean IKDC of 81.58% (range 76.3–91.3, SD 6.0), mean VAS of 21.30% (range 1.40–3.90, SD 1.0) and mean ROM of 137.70° (range 145.0–125.9, SD 6.4). The SB group reported a mean Kujala score of 86.62% (range 75.2–96.0, SD 5.6), mean Lysholm score of 86.67% (range 79.1–92.1, SD 4.3), mean Tegner score of 5.06 (range 4.0–5.6, SD 0.6), mean IKDC of 76.18% (range 68.9–82.3, SD 6.8), mean VAS of 20.50% (range 1.0–4.3, SD 2.3), and mean ROM 127.73° (range 117.2–141, SD 12.1). All these endpoints scored in favour of the DB group: Kujala (+ 3.2, *P* = 0.03), Lysholm (+ 5.1, *P* = 0.001), Tegner (+ 0.3, *P* = 0.2), IKDC (+ 5.4, *P* = 0.01), VAS (+ 0.8, *P* = 0.3), ROM (+ 9.96, *P* = 0.04). In the DB graft, a reduction of overall complications (OR 0.59; 95% CI 0.37–0.89 *P* = 0.1), further surgeries (OR 0.64; 95% CI 0.35–1.14; *P* = 0.12) and re-dislocations (OR 0.61; 95% CI 0.31–1.21; *P* = 0.16) was observed. The SB group reported a reduction in the post-operative apprehension test (OR 2.42; 95% CI 1.42–4.12; *P* = 0.24).

## Discussion

According to the main findings of this systematic review, the DB graft scored greater in terms of mean ROM, Kujala, IKDC, and Lysholm scores. Tegner score and VAS, further complications, revisions, and re-dislocations rate were remarkably in favour of the DB graft group, however, no statistically significance was found.

Recently, Kang et al. [72], performed a systematic review comparing SB versus DB using exclusively the hamstring tendon. They analysed 1116 knees (254 SB versus 862 DB), and focused on the Kujala score, apprehension test, re-dislocations, and overall complications. An increased risk of post-operative apprehension test in the SB group and of joint stiffness in the DB group was shown. No other relevant differences between the two grafts were detected. Lee et al. [73] performed recently a meta-analysis analysing the surgical techniques for patellofemoral instability. They reviewed even two clinical trials [12, 14] that compared DB vs SB, founding reduced instability, revisions and better clinical scores result in the DB group [73]. Two biomechanical studies [74, 75], comparing the two bundle methods, revealed that both reconstructions are able to restore adequate patellar stability. Placella et al. [75] stated that the ultimate load was 213 ± 90 N and 171 ± 51 N for the DB and SB, respectively. It was shown that the DB is more anatomical with better physiological stress distribution and, therefore, simulates the ultimate load more so than the SB graft [75], and better mimics the MPFL track at reduced flexion angles [74]. Furthermore, Wang et al. [74] found that the DB generates greater resistance to lateral displacement at the first 15° of knee flexion. Recently, Kang et al. [76] performed a systematic review of over 691 procedures comparing the techniques for patellar fixation in DB-MPFL reconstruction. They found a similarity between bone tunnel, suture anchors and suture techniques in terms of Kujala score, apprehension test, dislocation rate and further complications [76]. They stated that all these techniques achieve satisfactory patellar fixation for DB-MPFL reconstruction [76].

In the present systematic review, all scores of interests were remarkably in favour of the DB graft, with a good homogeneity of values in all comparisons. The range of values was narrow, especially for the Lysohlm score and IKDC. The standard deviation was small in all comparisons, detecting low data dispersion and feasible results. Similar observations were seen for the comparison of ROM, which reports a considerable improvement in favour of the DB graft. As such, these results are trustworthy and reliable. The Tegner score and visual analogic scale reported minimally improved values in the DB graft group. Data from these scores were reported by few studies and are not sufficient to draft reliable conclusions. Additionally, the level of significance according to the t-test is poor, detecting similarity between the techniques. Concerning the other outcomes of interest, no statistically significant result was obtained among all the comparisons. The *t* test detected marked similarity between the techniques in all the comparisons. However, the results of the comparisons of further surgeries and re-dislocations were considerably in favour of the SB graft. The analysis of the overall complications detected remarkable risk in the SB graft group, and the value of the *t* test was closer to the cut-off. All other comparisons detected minimal differences between the two groups.

Limitations of this work are several. First, most of the enrolled studies had poor level of evidence, being mostly retrospective. Few studies randomized samples, while none took advantage of blinding methods. Therefore, the overall quality was remarkably reduced, and data from this work must be interpreted with caution. Heterogeneous inclusion and exclusion criteria, along with the poor analysis reported by some of the enrolled studies, were other important limitations. Data analysis were performed regardless of the type of graft (autografts, allografts, synthetics). Further studies should be addressed to clarify the role of each grafts and related potential advantages. However, the comprehensive nature of the literature search, along with the strict eligibility criteria represent important points of strength of this systematic review. Furthermore, the good quality of the methodological assessment and the optimal baseline comparability decreased the risk of publication bias, improving the overall reliability of this work.

## Conclusion

The main findings of this systematic review support that a double bundle graft achieves statistically significant improvement in joint function in patients with patellofemoral instability who undergo MPFL reconstruction. Improvement was evidenced in the range of motion, Kujala, IKDC, and Lysholm scores compared to a single bundle graft.
