# Correction to: Single‑ versus double‑bundle patellar graft insertion for isolated MPFL reconstruction in patients with patellofemoral instability: a systematic review of the literature

**DOI:** 10.1007/s00402-021-04012-w

**Published:** 2021-07-19

**Authors:** Filippo Migliorini, Andromahi Trivellas, Giorgia Colarossi, Jörg Eschweiler, Markus Tingart, Björn Rath

**Affiliations:** 1 Department of Orthopaedics, RWTH Aachen University Clinic, Pauwelsstraße 30, 52074 Aachen, Germany; 2 Department of Orthopaedics, David Greffen School of Medicine at UCLA, Los Angeles, CA USA; 3 Department of Cardiosurgery, RWTH Aachen University Clinic, Aachen, Germany

## Correction to: Archives of Orthopaedic and Trauma Surgery (2020) 140:769–776 10.1007/s00402-020-03376-9

The article Single‑versus double‑bundle patellar graft insertion for isolated MPFL reconstruction in patients with patellofemoral instability: a systematic review of the literature, written by Filippo Migliorini, Andromahi Trivellas, Giorgia Colarossi, Jörg Eschweiler, Markus Tingart and Björn Rath, was originally published Online First without Open Access. After publication in volume 140, issue 6, page 769–776 the author decided to opt for Open Choice and to make the article an Open Access publication. Therefore, the copyright of the article has been changed to © The Author(s) 2021 and This article is licensed under a Creative Commons Attribution 4.0 International License, which permits use, sharing, adaptation, distribution and reproduction in any medium or format, as long as you give appropriate credit to the original author(s) and the source, provide a link to the Creative Commons licence, and indicate if changes were made. The images or other third party material in this article are included in the article’s Creative Commons licence, unless indicated otherwise in a credit line to the material. If material is not included in the article’s Creative Commons licence and your intended use is not permitted by statutory regulation or exceeds the permitted use, you will need to obtain permission directly from the copyright holder. To view a copy of this licence, visit http://creativecommons.org/licenses/by/4.0/.

The original article has been corrected.

Open Access funding enabled and organized by Projekt DEAL.

Funder Name: Universitätsklinikum RWTH Aachen (8915).

